# Temporal model for central sensitization: A hypothesis for mechanism and treatment using systemic manual therapy, a focused review

**DOI:** 10.1016/j.mex.2022.101942

**Published:** 2022-11-28

**Authors:** Adi Halili

**Affiliations:** Halili Physical Therapy 268 E River suite 130 Tucson AZ 85704

**Keywords:** Central sensitization, Systemic manual therapy, Chronic pain, Alzheimer's disease, Parkinson's disease, Ehlers-Danlos syndrome, Mast cell activation syndrome, Fibromyalgia, Whiplash associated disorders, Long COVID syndrome

## Abstract

The purpose of this focused review is to develop a consolidated hypothesis as to the causes and mechanisms of central sensitization and a related model for a treatment approach using Systemic Manual Therapy (SMT).

The key to understanding central sensitization is a firm grasp on structure and function of the Locus-coeruleus noradrenaline system (LC-NA). This system uses an elaborate switching mechanism to control the level and rate of activation of multiple systems.

This review evaluates the mechanisms and temporal relationships behind four components: salient stimuli, threat coding, aberrant afferent input, and oxidative stress.

The five-stage temporal model for central sensitization includes phasic activation of the LC-NA system, salient stimuli, threat coding of salient stimuli, central sensitization, and neural degeneration.

The three components of treatment include temporarily reducing afferent visceral input, shifting humoral inflammatory activity away from the brain and outside the body, and reducing oxidative stress by making oxygenated blood more available around the LC and other stressed areas in the brain.

The SMT protocols that could help in reduction of visceral afferent input are GUOU, Barral and LAUG. Protocols that should shift humoral inflammatory activity away from the brain or completely out of the body include UD and DCS. One protocol that can potentially reduce oxidative stress by making oxygenated blood more available around the LC is CCCV.

Future research and hypothesis-testing strategies as well as limitations are further discussed.

## Highlights

• This paper brings forward a consolidated comprehensive hypothesis for central sensitization.
• It describes central sensitization both as from a structural and a functional time dependent phenomenon.
• Based on that temporal model, a specific treatment method is proposed using Systemic Manual Therapy.

## Graphical abstract

**Figure:**
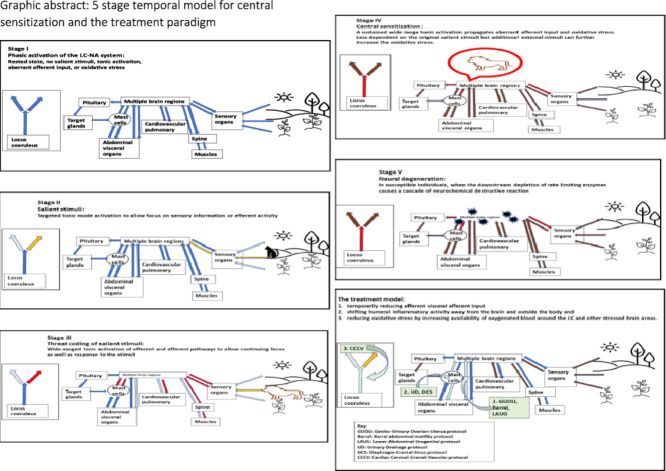
Graphical abstract

Specifications table

**Table:** 

Subject area:	Neuroscience
More specific subject area:	*Physical Therapy for central sensitization*
Name of reviewed methodology:	*Systemic Manual Therapy*
Resource availability:	*References and Datasets* 1. *Halili A. (2020), Systemic Manual Therapy. San Bernardino, Kindle direct publishing, California* 2. Halili, A. (2021a). Treatment for the central sensitization component of knee pain using systemic manual therapy dataset. *Mendeley Data, V1,* doi:10.17632/n7wrm2r3j6.1 3. Halili, A. (2021b). Physical therapy for the treatment of respiratory issues using systemic manual therapy protocols. *Journal of Bodywork and Movement Therapies, 27,* 113–126. https://doi.org/10.1016/j.jbmt.2021.02.009 4. Halili A. (2022), Application of HOAC combined with APD to develop physical therapy treatment for facial and jaw pain associated with trigeminal neuralgia using Systemic Manual Therapy (SMT) dataset, Mendeley Data, v1 *http://dx.doi.org/10.17632/759rh3ymdn.1*
Key words:	Central sensitization; Systemic manual therapy; Chronic pain; Alzheimer's disease; Parkinson's disease; Ehlers-Danlos syndrome; Mast cell activation syndrome; Fibromyalgia; Whiplash associated disorders; Long COVID syndrome*.*
Review questions:	1. What are the mechanisms of central sensitization 2. What treatment method should be effective in treating central sensitization 3. How the hypotheses for mechanism and treatment of central sensitization should be tested

##  

### Perspective

The temporal model presented expands on prior models for central sensitization allowing a more unified understanding of this phenomenon. Furthermore, it identifies the key element that can be targeted for treatment. Input to and from the LC-NA system is what drives the body's threat response, central sensitization appears to be dependent on rate and length of time. This review proposes a hypothetical treatment approach using systemic manual therapy.

## Introduction

The purpose of this focused review is to develop 1) a consolidated hypothesis as to the causes and mechanisms of central sensitization, and 2) a related model for a treatment approach using Systemic Manual Therapy protocols (SMT) [24].

CS is a term initially introduced by Woolf [76]. Early research focus was on the pathology of chronic pain and hyperalgesia [77]. These early studies described CS in terms of the morphological changes in afferent pathways associated with localized areas of persisting pain or pathology. Other works identified the existence of CS, or symptoms that are now associated with CS, in the presence of other conditions such as rheumatoid arthritis [45], osteoarthritis [39], Ehlers Danlos syndrome [16,50], as a sequala to whiplash injury [20], COVID-19 infection ([10,14]), or psychological trauma [47]. Although important, none of these studies established a cohesive comprehensive theory for CS. Recently we can see attempts to establish such hypotheses for central mechanisms and topography beyond the peripheral morphological changes [52,69]. The aim of this review is to further expand on that latter work and create a model that further ties together more of the components thought to be associated with CS. This model should allow for the future development of a treatment model. As a hypothetical example for such treatment, we explore the rationale behind the use several SMT protocols.

SMT refers to several dozen treatment protocols comprised of various osteopathic and manual physical therapy techniques such as fascial counterstrain (SCS) [68], Barral [6], integrative manual therapy (IMT) [73] and muscle energy techniques (MET) [46]. Individual techniques are grouped into protocols and performed in the exact same order each time. This standardization allows for methodical scientific evaluation of the effectiveness of SMT when treating various problems such as respiratory issues [26].

### The locus coeruleus-noradrenaline system

The key to understanding CS is to obtain a firm grasp on structure and function of the locus coeruleus-noradrenaline system (LC-NA) (also known as locus coeruleus-norepinephrine system). The locus coeruleus (LC) resides in the upper dorsolateral pons adjacent to the brain's fourth ventricle [57]. It projects a vast network of noradrenergic fibers with direct and indirect reach to all brain regions as well as the spine and other target organs in the body via hormonal or immunomodulated agents [54,57]. The LC-NA system also receives afferent input from multiple brain regions, the spine, and the immune and hormonal systems [23,42]. Because of this vast distribution, the LC-NA system plays a crucial role in regulation of several key functions including entrenching information into memory [8], threat detection [54], sleep/wake cycles [56], arousal [51,56], visceral function [60], hormonal control [42], immune regulation [42], the circulatory system [8], integration of sensory information [42], motor function [8], cranial nerve function [51] and modulation of pain mechanisms [62].

Activation or inhibition of GABA and glutamate by NA is the basic switching mechanism of the LC-NA system. The glutamate exerts an excitatory influence at the post-synaptic site, and GABA an inhibitory one [8,^42^,^54^,^56^,60]. A key point is that this interaction occurs both in efferent and afferent pathways [8,51].

Further refinement of this neuroregulatory mechanism is accomplished by having three types of adrenergic receptors: α1, α2 and β, each consisting of several subtypes. The α1 and β receptors are present primarily at postsynaptic sites and have an excitatory effect. The α2 receptors reside in both post- and presynaptic sites and have an inhibitory effect via a negative feedback loop [8,56].

Functionally, the LC-NA system behaves as an elaborate switchboard. It does so through two different modes of operation: phasic (1–6 Hz) or tonic (10–15 Hz) [30,72]. Phasic activation allows for more nuanced neurological function, while tonic activation occurs in response to novel or salient stimuli [30].

Tonic activation of the LC occurs with exposure to a number of mediators such as corticotrophin-releasing hormone (CRH) [42], histamine [33], acetylcholine (ACh) and nicotine [57]. This afferent activation is both neurogenic and humoral and employs mediators such as mast cells [59]. Both phasic and tonic activation are part of normal neurological function. However, persistent tonic activation is considered as the key factor contributing to central sensitization.

Initially, a persistent state of tonic activation occurs with multiple or intense salient stimuli that are perceived as a threat [9,30]. Over time however, this functional state could be preserved by both internally-sourced, aberrant afferent input ([29,42]) and persistent oxidative stress [32,^67^,79]. The combination of these last two factors is the essence of central sensitization in our model.

### Components of central sensitization

Before we can explore the treatment basis for central sensitization, we need to discuss in further detail the mechanisms and temporal relationships behind four components: salient stimuli, threat coding, aberrant afferent input, and oxidative stress.

**Salient stimuli** can be any sensory input that attracts our attention. This can occur when the LC-NA is in a phasic or tonic state. When a stimulus is perceived as salient, the LC-NA signaling facilitates glutamate release to activate pathways in need of attention and GABA release in ones that do not ([54,72]).

**Threat coding** of a salient input is done when corticotrophin-releasing factor (CRF) is released by several structures in the limbic system such as the bed nucleus of the stria terminalis, Barrington's nucleus, and the central nucleus of the amygdala into the LC or peri-LC region ([9,^51^,56]). This could occur in response to stimuli such as smell, sound, bright light, exposure to cold or hot temperature, physical or emotional trauma, any production of pain, or exposure to pathogens such as viruses, bacteria, or toxic substances ([9,^51^,56]).

***Aberrant afferent input*** to the LC-NA system comprises persistent salient stimuli which are considered threatening, and/or other internal afferent inputs from regions that are in a stressed state due to prolonged under-activation or overactivation by the LC-NA system. Some examples of this input include a sustained state of anxiety, altered sleep/wake status, depressed digestive, urinary and sexual function, altered cognitive processing, and overactivation of mast cells. A key point is that this aberrant afferent input from various regions can sustain the LC-NA in a state of frequent tonic activation regardless of the continuous presence or absence of the original salient stimuli ([29,42]).

One factor aiding the propagation of aberrant afferent input is the depletion of various rate-limiting compounds. We can indirectly observe these effects on several pituitary-derived hormones such as thyroid-stimulating hormone (TSH), reproductive hormones such as estradiol, and adrenocorticoids such as cortisol ([[9], [29], [59], [63], [65]). We can also see the decrease in levels of neurotransmitters such as acetylcholine [57], serotonin [8], and dopamine [56]. Initially the frequent tonic activation of the LC-NA system will facilitate overactivation of pathways controlled by these hormones or neurotransmitters, but over time, because of the partial or intermittent depletion of these rate-limiting compounds, it is the chronic under-activation of these previously overactive pathways that causes additional dysregulation and further magnifies the stressful afferent input acting on the LC ([29,42]).

***Oxidative stress*:** The LC-NA system is experiencing oxidative stress when it is in a persistent or frequent tonic state. There are a number of hypothesized components to the creation of oxidative stress on this system. One is that tonic activation requires more metabolic energy than a phasic one. The conversion process of dopamine to noradrenaline (NA) becomes less efficient, making less NA available to the LC-NA system [32,^67^,79].

However, the reduction in availability of NA does not, by itself, provide a sufficient explanation to many of the long-term neurological symptoms observed in sensitized patients. Therefore, we also need to consider several items: the adaptive plasticity occurring at the post-synaptic site that contains glutamate and GABA receptors; the functional and topographical differences between the α_2,_ β, and α_1_ adrenal receptors; and the role of several rate-limiting factors, discussed in the previous section.

Both β and α_1_ have a larger diameter than the α_2_ receptors, and they are primarily located at the postsynaptic site in contrast to the α_2_ which resides in the presynaptic site [8,56]. Because of these reasons, a scenario where availability of noradrenaline/norepinephrine is limited would favor the excitatory action at the postsynaptic over the inhibitory one at the presynaptic site.

In addition, with persistent input from α_1_ receptors, the glutamate system will increase α-amino-3-hydroxy-5-methyl-4-isoxazolepropionic acid receptor (AMPAR) and N-methyl-D-aspartate receptor (NMDAR) density and reactivity at the postsynaptic site, allowing for faster depolarization with less input from the LC-NA system [36,66]. Also, with the same tonic state, changes in β receptors on GABA-releasing postsynaptic sites result in long-term potentiation of these sites which in turn causes conversion of inhibitory cells to excitatory ones. [29]. So, while initially these changes in the glutamate-releasing site allow the brain to bias retrieval of external or internal information and execution of specific efferent function, over time, they contribute to the propagation of less bias in afferent and efferent information. Two clinical examples of this phenomenon are the hyperalgesia and allodynia seen in chronic regional pain syndrome (CRPS) [77] and facial-nerve synkinesis seen as a residual of viral facial-nerve palsy such as Ramsay-Hunt Syndrome or severe cases of Bell's Palsy [78].

As noted previously, another factor contributing to this afferent gain is the central modulation of mast cells. Mast cells are influenced by several external and internal factors. One central control and modulation mechanism is the balance between input of CRH (corticotrophin-releasing hormone) and input of cortisol. Considering the scenario discussed previously, where cortisol levels are being intermittently depleted, coupled with the remaining influence of CRH on the mast cells, they will act with greater vigilance, releasing more histamine into the peri-LC area and further contributing to the maintenance of tonic state activation ([21,^31^,^34^,^38^,65]).

To summarize this point, in a scenario where there is a system-wide sustained or frequent response to threat or stress coupled with the functional and topographical differences between the α_2_ and the β and α_1_ adrenal receptors, as well as uneven activation of immune modulators such as mast cells, the positive feedback loop that drives the state of oxidative stress and reduced availability of NA is further entrenched.

### Additional Considerations

***Self-reinforcing vs. repeated external stressors:*** Considering the morphological changes to the neurocircuitry discussed in this review [29,^36^,66] our model predicts that symptoms associated with CS can occur with or without an underlying pathology or disease process.

If this contention prevails, we should expect some variability in treatment outcome. Patients with an active on-going pathology such as, for example, collogen processing abnormality seen in Ehler Danlos syndrome (EDS) [16], would likely be more resistant to intervention than those with self-reinforcing sensitization without one. That resistant would be expected not necessarily because of the influence of the existing pathological process on pain, but rather it's contribution to the maintenance of the sensitized state.

Internalizing this dichotomy by the clinician, whether it is a primary care physician, a neurologist, a physical therapist, or other, could, on one hand, reduce the practice of simply dismissing patients with chronic pain conditions as a malingerer just because there are no available tests to explain their complaints [43]. On the other hand, once a successful treatment regimen for CS is identified, a failure to improve following treatment could be an added impetus for more focused pursuit of a previously missed active pathology.

***Central sensitization and neurodegeneration:*** There is a developing body of evidence ([5,^44^,^54^,^55^,79]) suggesting that in some individuals, this persistent state of sensitization would eventually contribute to a number of neurodegenerative conditions such as Alzheimer's disease (AD) and Parkinson's disease (PD). However, it is important to note that most individuals who are in a state of central sensitization, not develop a neurodegenerative condition [18]. If, however, the hypothesis discussed in this paper is reaffirmed, then central sensitization can be either reversed or at least controlled. An intriguing question is what would the outcome be if a targeted treatment for central sensitization is added to pharmacological interventions during the prodromal phase of AD or PD.

For our model to account both for a scenario where the aberrant afferent input and oxidative stress are reversible without significant neurological sequelae and one where more permanent neurological degeneration occurs (and is therefore not reversible), we need to consider some additional genetic vulnerabilities. These genetic factors, in the presence of oxidative stress, will cause actual structural deterioration beyond the persisting functional stress.

There are 3 gene mutations associated with early onset AD: amyloid precursor protein (APP), presenilin 1 (PSEN1), and presenilin 2 (PSEN2). Over 20 mutations are associated with late onset of the disease [22].

**Fig. 1:**
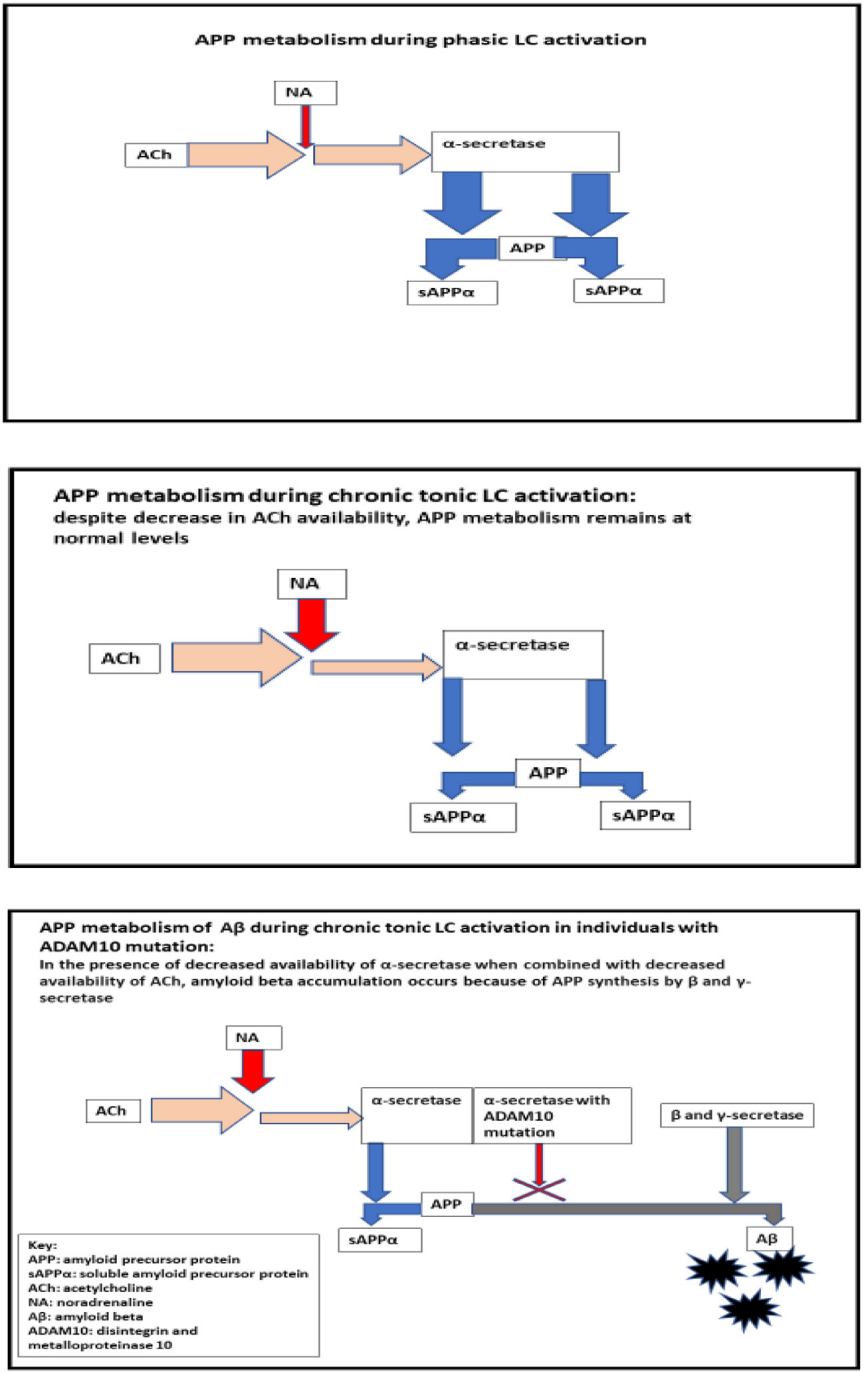
The relationships between Alzheimer's disease and central sensitization.

To illustrate how these mutations play a role in our model, let's discuss a scenario associated with a mutation of the α-secretase protein called disintegrin and metalloproteinase 10 (ADAM10). This protein is activated by Ach, and it is necessary for the processing of APP (amyloid precursor protein) via a non-amyloid pathway. When there is sufficient availability of α-secretase, the APP molecule will be processed to produce soluble amyloid precursor protein (sAPPα) which performs protective neuroregulatory functions. However, if α-secretase is scarce, the APP molecule will instead undergo cleavage by β-secretase and γ-secretase resulting in accumulation of amyloid beta (Aβ) as a byproduct [13,^49^,80]. As Aβ accumulates, further accelerated neurodegeneration that is independent of the prior metabolic sequelae would take place [55]. In the previously discussed scenario, frequent tonic activation of the LC-NA causes reduced availability of ACh, which in turn causes reduction of overall availability of α-secretase (which is already limited because a portion of it is in its nonfunctional mutated form, β-secretase.) The result is that the γ-secretase Aβ pathway becomes prominent. Our model predicts a similar scenario in the presence of other mutations, where there is an underlying shortage of an essential protein due to a mutation, but this shortage would not be functionally expressed until additional metabolic stress is introduced by reduced availability of ACh. Fig. 1 illustrates this process.

The underlying pathological mechanism for PD is even more complicated. While acknowledging the fact that there are several subtypes of PD. But to illustrate the relationship between the genetic vulnerability and central sensitization, as an example, we will discuss the familial form of PD that is associated with several mutations of the SNCA gene such as A30P and A53T [4,79]. These mutations lead to overproduction of a protein called α-Synuclein (α-Syn). Although PD is associated with neurodegeneration of dopamine-producing neurons and the aggregation of α-Syn which forms plaque deposits called Lewy bodies ([4,^11^,79]). We still need to consider that α-Syn normally has a protective function and facilitates the recycling of synaptic-vesicle dopamine, bringing it back into the neuron from the extracellular space. We also need to consider that PD generally develops during late stages of life despite the presence of the over-expression of α-Syn from birth.

Because of these two inconsistencies, and to be able to consolidate the pathogenesis of PD with our central sensitization model, we need to consider the following additional factors: In addition to the role α-Syn plays in vesicle recycling, it also has a less-recognized function of limiting the availability of the rate-limiting enzyme tyrosine hydroxylase (TH). TH is used in the conversion of L-Dopa to dopamine [79]. Under normal circumstances, when there is a limited availability of TH, dopamine is also produced (as a byproduct) by alternate enzymatic processes/pathways; these processes also generate reactive oxygen species (ROS) [67]. Those same enzymatic pathways are also used when there is oxidative stress created by increased demand for production of NA from dopamine [32].

One can consider a complex scenario where this secondary enzymatic pathway is already more prominent because of the overabundance of α-Syn, as in individuals who carry the A53T mutation. Moreover, in tonic activation of the LC during a sustained stress response, there is an additional demand on production of dopamine to satisfy the upstream requirement to convert it to NA.

**Fig. 2:**
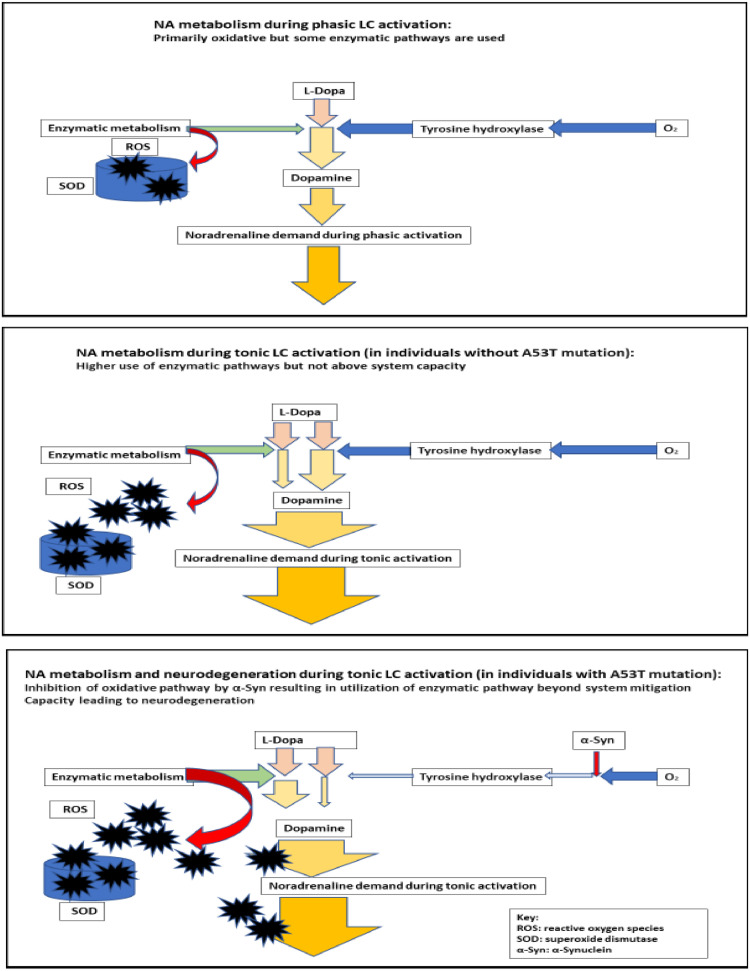
The relationships between Parkinson's disease and central sensitization.

It is the aggregation of these two factors that causes the levels of ROS to rise beyond the self-regulatory capability of several superoxide dismutase (SOD) subtypes. SOD normally converts ROS to other, less reactive agents [67], but with ROS rising to such high levels, the ROS start to bind with other structures such as α-Syn, provoking neurodegeneration [4,79]. As is the case with accumulation of Aβ in AD, the formation of Lewy bodies is likely to further escalate the cascade of destruction, independent of the original conditions leading to the production of ROS. This process is illustrated in Fig. 2.

### The temporal model

To understand how the factors discussed previously (salient stimuli, threat coding, aberrant afferent input, and oxidative stress) interact over time, we developed the five-stage temporal model for central sensitization.

**Stage one** denotes a phasic activation of the LC-NA system. During **stage two**, the system shifts into tonic mode, activating specific regions as a response to salient stimuli. During **stage three**, the stimuli are coded as a threat which results in more wide-ranging tonic activation. **Stage four** is entered after a prolonged and sustained period in stage three. At this stage, the stress response is less dependent on external stimuli and is propagated by the sustained aberrant afferent input and a chronic state of oxidative stress. Also, in this stage, a salient stimulus that was not previously considered as a threat might still trigger a stress response. **Stage five** would be entered, in susceptible individuals, when the downstream depletion of rate-limiting enzymes causes a cascade of neurochemical reactions leading to neural degeneration.

The neurophysiology attributed to the first three stages is not considered pathological, but it is necessary to describe these stages in order to be able to explain how the neurological system reaches stage four and five. Our treatment model is focused on stage four, since, at earlier stages, a less complicated, already-existing treatment approach might be as effective, and at stage five, we anticipate that the advanced degree of neural degradation would make any approach less efficacious without additional pharmacological manipulation Table 1.

**Table 1:** 5-Stage Temporal Model for Central Sensitization.

Stage	Activity	Description	Comments
I	Phasic activation of the LC-NA system	Resting state, no salient stimuli, tonic activation, aberrant afferent input, or oxidative stress	Normal functional state
II	Salient stimuli	Targeted tonic mode activation to allow focus on sensory information or efferent activity	Normal functional state
III	Threat coding of salient stimuli	Wide-ranging tonic activation of efferent and afferent pathways to allow continuing focus as well as response to the stimuli	Normal functional state
IV	Central sensitization	A sustained wide-ranging tonic activation propagates aberrant afferent input and oxidative stress. Less dependent on the original salient stimuli but additional external stimuli can further increase the oxidative stress.	Abnormal functional state
V	Neural degeneration	In susceptible individuals, when the downstream depletion of rate-limiting enzymes causes a cascade of neurochemical destructive reactions	Neuropathological state

### The topography

While keeping in mind the physiological model previously described that included the LC and a loop containing an efferent and afferent limb, as well as the adaptations described in the temporal model, we need to also define our anatomical therapeutic targets. In addition to the locus coeruleus, the regions associated with the stress response are the amygdala [28,^42^,56], prefrontal cortex [23,28], hippocampus [37], hypothalamus [3,51], pituitary [3], spine and cranial nerves [39,^51^,^57^,^58^,62], cardiovascular system ([7,^8^,^48^,^51^,^56^,60]), visceral system [8,^56^,60], and the hormonal and immune systems [3,^21^,^31^,54].

When we tie this topography to impairments or conditions associated with these regions, we should be able to evaluate the effectiveness of treatment on cognitive function ([28,64]), anxiety ([2,^23^,42]), depression [35], insomnia [8], fatigue [8], pain [8], digestive [8], urinary and sexual visceral dysfunction [40], immune and hormonal behavior ([42,59]) and motor function [8], cranial-nerve-associated conditions [35], and migraines and other types of headaches [2,^41^,71].

The degree of success of the treatment model on each of one of these impairments can help us test both the temporal model for CS and the proposed treatment strategy.

### The treatment model

When considering a comprehensive treatment approach for central sensitization we need to consider that a sustained sensitized state could be a product of several simultaneous factors. Rather than a progressive disease process, our model suggests that while sensitization often occurs in the presence of disease or dysfunction, it is a distinct entity. The second key factor is the temporal element of sensitization. We need to consider not just the intensity, but also the rate of stress on the LC. The final element for consideration is the summative nature of that stress.

Considering these three factors we can postulate the following:

1. The treatment approach should be effective, although to a lesser degree, even in the presence of an underlying disease or neurodegenerative process.
2. An attractive objective of treatment is to reduce the rate of input toward the LC. Even if we can only temporarily reduce participation of a given element in the sensitization process, if we can do so with sufficient frequency, the reduction of the overall rate could shift the LC persistent state from tonic to phasic.
3. Because sensitization is maintained by multiple contributories, we could achieve reduction of input rate by incorporating different treatments to address several systems rather than looking at one pathway, such as the approaches taken with vagal nerve stimulation (VGS) [27], hormonal supplementation [74] or a single-agent treatment such as gabapentin [19]. This concept is valuable considering patient variability. It is possible that in one patient the visceral loop is dominant while in another it is a spinal pain pathway etc. Having more than one target area should allow for broader clinical success.
4. When considering specific interventions, focus should be on three elements: temporarily reducing afferent visceral input, shifting humoral inflammatory activity away from the brain and outside the body, and reducing oxidative stress by making oxygenated blood more available around the LC and other stressed areas in the brain.

There are number treatment methods that are likely to reduce the overall neurological afferent gain toward the LC. These approaches are expected to address the visceral system, alter inflammatory presentation, and manipulate the vascular system. Among the methods that can be considered, they either demonstrated these types of effects or are at least hypothesized to do so. These methods include strain counterstrain and fascial counterstrain ([15,^68^,^69^,75]), Barral visceral and neuro mobilization techniques [1,^6^,81], integrative manual therapy [17,73], craniosacral therapy [53,70], positional release techniques [12,61], and systemic manual therapy (SMT) [24].

While all the methods listed above consists of some techniques that can address the systems involved in sensitization, only SMT is practiced in a manner that allows for systematic evaluation of large datasets. As discussed previously, while SMT protocols contains techniques that are originated from fascial counterstrain, integrative manual therapy, Barral, and others, the standardized manner of application ensure that a specific system and anatomical regions are treated with a given protocol. Therefore, as long as the internal validity threats are controlled [25], one can potentially observe the effects associated with desensitization on a problem remote from the treated area or system. For example, if improvement in knee pain is noted when using a cranial protocol such as Cardiac-Cervical-Cranial-Vascular (CCCV) protocol, it can be attributed to desensitization and not local or regional effects.

Evaluating the outcomes noted with previously published work [26], there are several similar protocols that could help in reduction of visceral afferent input: Genito-Urinary-Ovarian-Uterus (GUOU), Barral Abdominal Motility (Barral) and Lower-Abdominal-Urogenital (LAUG). GUOU uses IMT techniques, LAUG comprises fascial counterstrain, and specific abdominal visceral motility techniques are used in the Barral protocol. Looking at the same work we can also find a couple of protocols that should shift humoral inflammatory activity away from the brain or outside the body: Urinary-Drainage (UD) and Diaphragm-Cranial-Sinus (DCS). We also find at least one protocol that can potentially reduce oxidative stress by making oxygenated blood more available around the LC: Cardiac-Cervical-Cranial-Vascular (CCCV).

### Hypothesis testing

Future studies will have to evaluate the individual problems associated with sensitization listed previously. For these studies to reject the null hypothesis, they will have to meet the following four conditions:

1. The study will have to demonstrate average improvement across episode of care in overall symptoms as well as in the specific problem studied (for example, knee pain, migraines, anxiety etc.). While improvement across episode of care does not indicate why it occurred, it does tell us the effects were durable.
2. We will need to find protocols associated with the treatment principles for desensitization (reducing afferent visceral input, shifting humoral inflammatory activity away from the brain and outside the body, and reducing oxidative stress) in the group that passes the Halili Physical Therapy Statistical Analysis Tool (HPTSAT) null rejection criteria (this refers to the criteria used to determine that an intervention or group of interventions is better in a clinically substantial way than the average of all other intervention combinations). The HPTSAT is a tool that uses a modified adaptive platform design to isolate the rate of improvement after a given intervention compared to all other interventions [25].
3. At least one of the protocols associated with reducing afferent visceral input (Barral, LAUG, GUOU), and one associated with shifting humoral inflammatory activity (UD, DCS), and CCCV protocol which is associated with reducing oxidative stress will be in this group regardless of whether they have proposed direct effect on the specific problem studied or not. For example, if we study how irritable bowel syndrome changes after treatment, we might consider that visceral abdominal protocols such as LAUG might cause an improvement because of a direct effect on the digestive system, however, to reject the null hypothesis, we will also have to identify other protocols associated with desensitization that are not considered to have a direct effect on the digestive system such as CCCV and DCS.
4. In each study evaluating an individual problem (such as back pain, migraines, dizziness etc.), we also need to see that the protocols associated with desensitization are passing the HPTSAT criteria for overall improvement. This condition is in place because our model considers sensitization a systemic phenomenon, and if the treatment indeed improves sensitization, the predicted improvements should occur in more than one criterion or problem.

Although not part of the hypothesis-testing process, we should also, in each of these proposed investigations, identify beneficial interventions associated with direct effects on the individual problem studied, and additional interventions that could affect sensitization but are not part of our original group of protocols. This additional focus could help further refine the pathological and treatment models for sensitization and identify effective treatments for a number of conditions that are generally difficult to treat.

### Discussion and conclusion

The temporal model developed in this review expands on existing understanding of CS while introducing several concepts. It identifies the differences and similarities between normal attention, threat coding, stress response, abnormal sensitization, and neurodegeneration processes. It defines central sensitization as a physiological state of frequent tonic activation of the LC-NA system, which is propagated by self-sustaining neural and humoral loops, cellular adaptation that causes a positive feedback loop, and failure of rate-limiting enzymes due to oxidative stress. The model also provides a common mechanism that can account for symptoms associated with several complex conditions such as fibromyalgia, whiplash associated disorders, post-traumatic stress syndrome, Ehlers-Danlos syndrome, mast cell activation syndrome, and most recently what is colloquially referred to as long COVID. But to the greatest degree, the most important contribution of this model is the recognition that CS is maintained by the rate and intensity of the self-sustaining loops into the LC-NA system. If this concept survives scrutiny, the dependency on rate and intensity is an Achilles’ heel that could be targeted by a viable treatment strategy for CS and by extension to all the conditions associated with it.

The treatment hypothesis contends that reversal or reduction in the severity of sensitization can be achieved with regular application of treatment protocols if they disrupt the self-propagated neural and humoral loops and reduce oxidative stress.

While number of treatment approaches could be effective in disrupting these self-sustaining feedback loops, the treatment model proposed in this paper promotes the use of use of SMT protocols over others. It does so not necessarily because these protocols are better than other approaches, but because unlike other methods, the manner of intervention is standardized. This standardization should allow to rigorously evaluate specific intervention and further refine the approach.

Future research should focus on validation of the model. This can be accomplished by a series of observational studies evaluating treatment effectiveness on several of the impairments this paper identified as associated with CS. If treatment uses SMT protocols, in order to reject the null hypothesis and support the temporal model hypothesis, four conditions to be met are outlined. These conditions include demonstration of measurable improvement of the specific impairment studied as well as overall improvement over episode of care, demonstration that the improvement occurs after treatment with the protocols associated with the desensitization process, demonstration that these protocols are effective even when the problem treated is anatomically or physiologically remote from where a specific protocols is applied, and the last condition, demonstrate that these protocols are effective not just treating the individual problem but demonstrate benefit for overall progress.

Additional empirical research is also needed to gain further understanding of the physiological effects of treatment and the pathophysiology associated with CS.

Another intriguing research question is what role could be served by a successful desensitization model in an overall treatment scheme for neurodegenerative conditions such as AD and PD.

### Limitations

• This review is intended to create the pathological basis and establish an intervention theory for central sensitization. It is intended to lay out the groundwork for future studies; it is not being presented either as a standalone model or a standalone treatment.
• Although comprehensive, it is not known if there are additional elements contributing to CS. Future research is needed to identify these additional elements and evaluate if changes to the model are necessary.
• The treatment model is not necessarily intended to identify the best treatment, but rather identify types of interventions that address central sensitization and are in the group that was found to be more effective than the oSOC (passing the HPTSAT criterion).
• The exact mechanism of treatment is not yet fully established. However, the existing understanding of treatment mechanisms is sufficient to at least test the hypothesis presented for the temporal model for CS.

## Ethics statements

The Author had followed MethodsX ethical guidelines, this work does not involve human subjects, animal experiments or data collected from social media.

## CRediT author statement

Adi Halili is the sole author and responsible for all elements of this paper.

## Declaration of Competing Interest

The authors declare that they have no known competing financial interests or personal relationships that could have appeared to influence the work reported in this paper.

## Data Availability

No data was used for the research described in the article.
